# Thinprep plus Papanicolaou Stain Method Is More Sensitive than Cytospin-Coupled Wright Giems Stain Method in Cerebrospinal Fluid Cytology for Diagnosis of Leptomeningeal Metastasis from Solid Tumors

**DOI:** 10.1371/journal.pone.0122016

**Published:** 2015-04-07

**Authors:** Zhenyu Pan, Guozi Yang, Yongxiang Wang, Hua He, Xiaochuan Pang, Yan Gao, Weiyan Shi, Yu Li, Lihua Dong, Yuanyuan Song

**Affiliations:** 1 Department of Radiation Oncology, the First Hospital, Jilin University, Changchun, China; 2 Department of Clinical Laboratory, the First Hospital, Jilin University, Changchun, China; 3 Cancer Center, the First Hospital, Jilin University, Changchun, China; BIDMC, UNITED STATES

## Abstract

**Background:**

The present study was designed to determine whether the Thinprep plus Papanicolaou stain (Thinprep) method is more sensitive than the Cytospin-coupled Wright-Giemsa (WG) stain (Cytospin) method in diagnosis of leptomeningeal metastasis (LM) from malignant solid tumors in cerebrospinal fluid (CSF). We also explored if the Thinprep method could be used in the differential diagnosis of the type of primary tumor cells based on the morphology of tumor cells in CSF samples.

**Methods:**

The morphological features of tumor cells in fresh CSF samples were analyzed using both methods. The tumor cell detection rates were compared between the two methods.

**Results:**

Using the Thinprep method, we found that each type of tumor cells in the CSF samples had specific identifiable morphological features linked to their primary cancer origins, such as adenocarcinomas originated from the lungs, breast, and stomach, and lung squamous cell carcinomas, small cell lung cancer, large-cell neuroendocrine lung cancer, hepatocellular carcinoma, and malignant melanoma. In a retrospective study with 88 LM patients, cancer cells were detected in 80 out of the 88 CSF samples. In the comparative study with 45 LM patients, the initial detection rate of the Thinprep method was significantly higher than that of the Cytospin method (73.3% vs. 57.8%, P<0.01). The cell morphology was better preserved and subcellular structures were clearer using the Thinprep method, compared to the Cytospin method.

**Conclusions:**

The Thinprep method is more sensitive and suitable for LM diagnosis in CSF in patients with malignant solid tumors than the Cytospin method. The Thinprep method may facilitate primary tumor detection and help design early treatment regimens for LM patients with tumors of unknown primary origin.

## Introduction

Brain metastasis is one of the most devastating clinical manifestations of advanced human cancers. The pathogenesis of brain metastasis remains unclear and the prognosis is poor for most cancer patients with brain metastasis. As one of the most dangerous of brain metastases, leptomeningeal metastasis (LM) is referred to as a condition with a wide spread of cancer cells to the subarachnoid space and diffuse infiltration to the pia or arachnoid mater [1]. Since the clinical manifestations of LM are often complex and non-specific, its diagnosis often depends on the findings from neuroimaging and cytopathology [1]. It has been suggested that gadolinium-enhanced MRI may be more sensitive than cytology, but its lower specificity precludes it from replacing cytology as the gold standard for diagnosis [2]. Therefore, an accurate diagnosis of LM requires the detection of specific malignant cells in the cerebrospinal fluid (CSF) [3,4].

The thin-layer preparation (ThinPrep) is a liquid-based cytology method and has been suggested to have an application in the diagnosis of LM using CSF samples [5]. During the ThinPrep analysis, the cells in CSF are collected through high-precision filtration driven by fluid mechanics and gently adsorbed onto a glass slide by using electrochemical forces. This ensures almost all the cells in a CSF sample to be collected and transferred onto the slide in an automated fashion. As one of the advantages over other methods, the cells are handled with minimal external disturbances during the collection process and therefore cellular and subcellular structures are well preserved. In comparison, the conventional cytocentrifuge (Cytospin) cell preparation followed by Wright-Giemsa (WG) stain (Cytospin-WG) is a well-established method commonly used in CSF cytology. Several published studies demonstrate that this method is appropriate for cytological examination of CSF in patients with LM from solid tumors [3,6]. Although the ThinPrep has been suggested to be better than Cytospin in analysis of CSF [5] and other non-gynecological specimens, [7–9] the former does not appear to be superior to the latter when used to analyze pleural effusion specimens [10].

Considering that there are no reports on direct, side-by-side comparison between the ThinPrep and Cytospin methods in the diagnosis of LM using CSF samples, we carried out a prospective clinical study to directly compare the sensitivity and specificity of the two methods in the diagnosis of LM in patients with metastatic solid tumors, using CSF specimens.

## Materials and Methods

### Ethics Statement

The study was established according to the ethical guidelines of the Helsinki Declaration and was reviewed and approved by the Ethics Committee of Norman Bethune First Hospital, Jin Lin University, Changchun, China. Each of the patients provided written informed consent before entering the clinical study. The informed consent form is administered in Chinese language. In the case of patients who could not read standard Chinese, a verbal consent was documented using the following procedure: the investigator or research nurse provided oral explanation of the purpose and the entire procedure of the study to the patient and provided answers to any questions that the patient had raised; the patient provided verbal consent which was recorded in the patient’s chart, including the contents of the discussion, the consent, the time and date; and an independent investigator or research nurse witnessed the verbal informed consent process by signing the records. This procedure was reviewed and approved by the same aforementioned Ethics Committee. All the informed consent forms were kept in the patients’ hospital charts.

### Clinical Characteristics of LM Patients in Retrospective Study

In this report, we will describe two studies: a retrospective study and a prospective study. In the retrospective study, there were a total of 88 consecutive LM patients with cancers of non-neurogenic and non-hematologic origins admitted to our hospital from August 2009 to October 2013. All patients underwent CSF cytological analysis by the Thinprep method (1–6 tests per patient) in our hospital. The patients were 31–72- years old (average age, 54 years), including 44 females and 44 males. Of the 88 patients, 71 had a history of confirmed malignancy, including lung adenocarcinoma (31), small-cell lung cancer (19), breast cancer (12), lung squamous cell carcinoma (3), gastric adenocarcinoma (3), breast and lung cancer (1), large-cell lung cancer (1), and hepatocellular carcinoma (1); the remaining 17 were initially diagnosed with LM and primary tumors were further identified in 15 of them, which included lung adenocarcinoma (10), gastric adenocarcinoma (2), small-cell lung cancer (2), and melanoma (1). Primary tumors of the other two patients were not identified and listed as unknown primary tumors. The diagnoses of the aforementioned cancers were confirmed by histopathological examination of primary tumors. The CSF samples were collected by lumbar puncture with a spinal needle being inserted between the lumbar vertebrae L2/3, L3/L4, or L4/L5.

### Clinical Data Collection

#### Clinical symptoms

The neurological symptoms and signs commonly associated with LM were observed and recorded in the following categories: 1) brain: dizziness, headache, vomiting, psychiatric symptoms and sleepiness; 2) cranial nerves: tinnitus, diplopia or blurred vision, palsy, and facial numbness; and 3) nerve roots: physical and sensory dysfunctions and urinary disorders. Patients having a combination of the above symptoms were also identified.

#### Magnetic resonance imaging (MRI)

All 88 patients underwent standard and enhanced brain MRI scans (1.5/3.0 T). In addition, 51 patients also had spine MRI scans. MRI results showed that 42 patients had pia mater/ependymal enhancement and metastatic nodules on the spinal cord or cauda equine, and 53 patients had metastatic nodules in the cerebral sulci or gyri, communicating hydrocephalus, and cerebral or spinal dura enhancement. Eleven patients had negative MRI results. Most patients with positive MRI results showed mixed imaging features.

#### CSF biochemical tests

Clinical chemistry tests were conducted for all the CSF specimens. Of the 88 patients, 78 had elevated protein (> 0.45 g/L) and 41 had decreased glucose (< 2.3 mmol/L) with eight having results within the normal ranges.

#### CSF cytological analysis

CSF specimens (7–14 mL) from all 88 patients were examined using the Thinprep method (1–6 tests per patient). Three cytopathologists independently performed the microscopic examination of all samples. Test results were presented as positive or negative. A positive result was defined as follows: 1) malignant cells were detected or 2) the cells exhibited some features of malignancy or were highly suspicious for neoplasm and the patient had a history of confirmed malignancy.

### Clinical Diagnosis of LM

The diagnosis of LM was made according to the National Comprehensive Cancer Network (NCCN) guidelines and American Society of Clinical Oncology (ASCO) guidelines. [11] The diagnosis for LM was made if any one of the following criteria was met: 1) tumor cells were detected in CSF cytological examination; 2) patient had a history of malignancy and the enhanced MRI scans showed diagnostic imaging features of LM, including focal or diffuse leptomeningeal enhancement, ependymal enhancement, and/or metastatic nodules on the spinal cord or cauda equine; 3) patient had a history of malignancy, exhibited LM-related neurological symptoms and the enhanced MRI scan showed imaging features suggestive of LM, including dura mater enhancement, metastasis in subarachnoid nodules, ventricular or parenchymal enhancing nodules, sulcal, folia, or cranial nerve enhancement, and/or communicating hydrocephalus; in addition, the CSF biochemical tests showed abnormal results such as elevated protein or decreased glucose levels, and patient had no history of traumatic brain injury or meningitis caused by pathogenic microorganisms; and 4) Patient had a history of malignancy and exhibited persistent or severe and progressive neurological symptoms typically associated with LM and the MRI scan was negative or inconsistent with the severity of manifestations; and patient had no other diseases that may cause such symptoms and LM-directed treatment effectively reduced the existing symptoms.

Eighty of the 88 patients were diagnosed with LM based on detection of malignant cells in the CSF by the Thinprep method. Of the eight patients with negative CSF results, five patients with small-cell lung cancer exhibited diagnostic imaging features of metastatic spinal tumor and two patients with lung squamous cell carcinoma. One patient with lung adenocarcinoma had symptoms such as severe persistent headache, vomiting and dizziness, elevated protein level in CSF, and imaging features characteristic of metastasis in the cerebral sulci or gyri. Patients had no other diseases that may cause such neurological symptoms. LM-directed treatment effectively reduced the existing symptoms.

### Comparative Prospective Study

#### Study subjects

Among the aforementioned 88 LM patients, 45 patients (24 females and 21 males) who underwent intrathecal chemotherapy were enrolled in the prospective, comparative study. These patients were 46–65-years old (average age, 53 years). Among them, 39 had a history of malignancy, including lung adenocarcinoma (21), lung squamous cell carcinoma (2), small-cell lung cancer (7), breast cancer (8), and gastric adenocarcinoma (1). The other six patients were initially diagnosed with LM and their primary tumors were then identified as lung adenocarcinoma (3), gastric adenocarcinoma (2), and small-cell lung cancer (1).

#### Sample collection

During the initial intrathecal chemotherapy, CSF sample (12–14 mL) from each of the patients was collected by lumbar puncture before injection of the chemotherapeutic agents (12.5–15 mg of methotrexate and 5 mg of dexamethasone). Each CSF sample was divided into two equal aliquots and immediately subjected to cytological examination by the Thinprep and Cytospin-WG methods.

#### Slide preparation, staining, and examination

For the Thinprep method, the CSF samples were added to 10 ml PreservCyt cell preservation solution, mixed, and allowed to stand for 15 min. Slides were prepared using the ThinPrep 2000 automated slide processor (Hologic, Bedford, MA, USA), fixed in 95% ethanol for 15 min, and stained by standard Pap method, following the manufacturer’s instructions. For the Cytospin-WG, the CSF samples were divided into three equal aliquots and loaded on to the Cytospin 4 cytocentrifuge (Thermo Scientific, Waltham, MA, USA). Cells were placed onto 3-aminopropyltriethoxy-silane-treated glass slides by centrifuging at 800 rpm for 3–5 min. The slides were allowed to air-dry for 10–15 min before staining with WG stain solution for 10–15 min, following the standard protocol of the manufacturer. The CSF slides were examined and tumor cells were detected as described above.

#### Statistical analysis

Data analysis was performed using SPSS 17.0 software. Positive predictive rates were compared using the paired McNemar’s test. P-values less than 0.05 were considered statistically significant.

## Results

### The Thinprep results from the retrospective study

**Fig 1:**
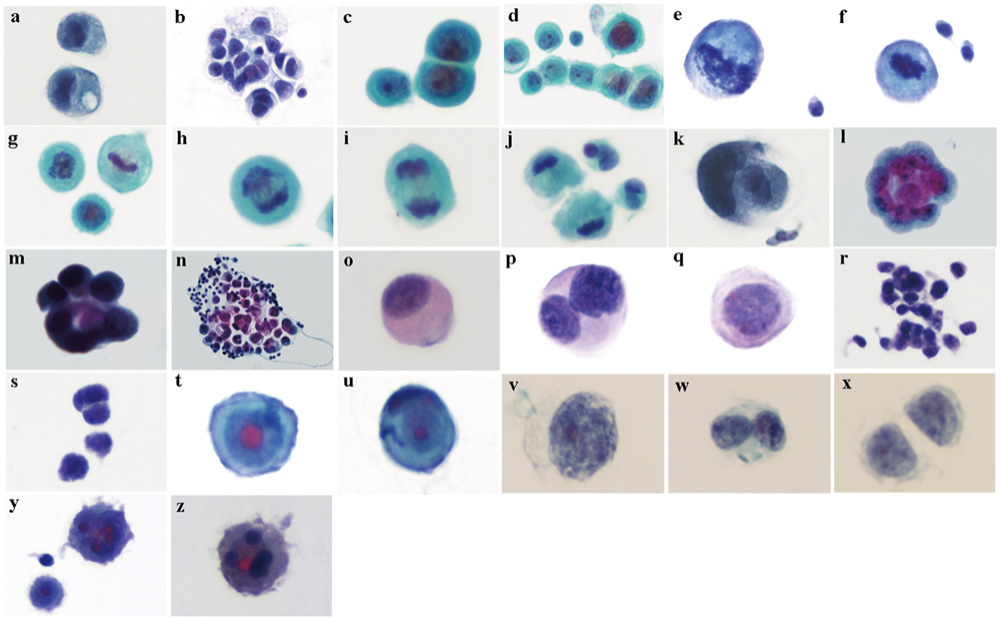
Different pathological types of tumor cells detected in CSF specimens. a-n, adenocarcinoma cells; o-q, cancerous squamous cells; r-s, small-cell lung cancer cells; t-u, hepatocellular carcinoma cells; v-x, large-cell lung cancer cells; y-z, malignant melanoma cells. (Thinprep, Pap staining, ×400)

All the 88 patients underwent several of CSF cytological examinations (1–6 times) by the Thinprep method in our hospital. Cancer cells were detected in 80 out of the 88 CSF samples. As shown in Table 1, of the 80 patients with positive CSF results, primary tumors were identified in 78 patients, including 58 adenocarcinomas,16 small-cell lung carcinomas, 1 large-cell lung carcinomas, 1 squamous cell carcinoma, 1 malignant melanoma, and 1 hepatocellular carcinoma. Primary tumors of the other two patients with positive CSF results were not identified. The Thinprep method preserved cytomorphological features with clear background, allowing differentiation of malignant cells of different origins based on their distinct morphological characteristics (Table 1, Fig. 1).

**Table 1:** Morphological features of different types of tumor cells in CSF specimens (n = 78, samples from all 80 LM patients had positive CSF results except two patients with unknown primary tumors).

	Adenocarcinoma n = 58	Squamous cell carcinoma n = 1	Small-cell lung cancer n = 16	Hepatocellular carcinoma n = 1	Large-cell lung cancer n = 1	Malignant melanoma n = 1
**Cell morphology**						
distribution	scattered or clustered	scattered	mostly clustered	mostly scattered	scattered or clustered	clustered or scattered
size	large, varying	large, varying	several times that of lymphocytes, varying	large, varying	large, varying	large, varying
shape	round or irregular	mostly round or oval	irregular	mostly round	mostly round or oval	mostly Round
edge	mostly smooth	smooth	mostly smooth	mostly smooth	not clearly defined	pseudopodia-like protrusions
**Cytoplasm**						
nucleo-cytoplasmic ratio	increased	increased	extremely high	significantly increased	extremely high	significantly increased
staining	blue, uneven	purple, pink in keratinized areas	diminished cytoplasm, faint staining	blue,uniform	diminished cytoplasm, faint staining	blue, uniform
vacuoles/granules	vacuoles of varying sizes	not observed	not observed	not observed	not observed	large, dark particles
**Nucleus**						
shape	round or irregular, lobulated, occasionally mitotic	round or oval, occasionally lobulated	irregular	mostly round, occasionally lobulated	round or irregular	mostly round
size/location	varying/ in the center or towards the edge	large/in the center or towards the edge	large/ in the center	mostly large/towards the center or edge	large/ in the center	large/mostly in the center
number per cell	single or multiple	single or multiple	single	single or multiple	mostly single	single or multiple
border	mostly smooth	smooth	Partially unsmooth	smooth	smooth	smooth
chromatin	densely packed, intensely stained	chromatin flocculationor coarse granular, intensely stained	chromatin, intensely stained	purple, uniform	coarse granular chromatin, intensely stained	intensely stained
**Nucleolus**	observed in some cells	observed in most cells	not observed	observed in most cells	observed in some cells	observed in most cells
staining	red or deep blue	red		red	red	red
size	varying	mostly small		mostly large, varying	mostly small	mostly large, varying
shape	round or irregular	round		round or oval	round	round or irregular, partial boundaries not clearly defined
number per nucleus	single or multiple	mostly multiple		Single in each nucleus	single	single or multiple

As shown in Fig. 1a-z, cells examined by the Thinprep method appeared intact, with good preservation of three-dimensional (3D) structures, while cellsprocessed by the Cytospin-WG method appeared shrunk, wrinkled, or deformed. The Thinprep method provided a clearer presentation of subcellular structures and layers, compared to the Cytospin-WG method. In particular, the structures such as nucleus, nucleolus, and chromatin were much more visible with the Thinprep method, allowing a more accurate detection and differentiation of tumor cells. In the Cytospin-WG method, a higher number of cells appeared ruptured or degenerated, and the nucleolus and chromatin structures were rather obscure, making it difficult to detect morphological features of malignant cells or to define their primary origins.

The morphological differences between pathological cell types were mostly observed in subcellular structures including nuclei, chromatin, nucleoli, and vacuoles. The major presentations are as follows:

1. Adenocarcinoma (Fig. 1a-n): scattered (Fig. 1a) or clustered distribution (Fig. 1b); intensely stained cytoplasm with occasionally observed vacuoles (Fig. 1a); most cells had intensely and unevenly stained chromatin and some showed fine granules; most cells had an irregular number of nucleoli, which were irregular in size and stained deep-red(Fig. 1a/b/c/d); some cells showed abnormal nuclear division(Fig. 1e/f/g) and cytoplasmic bridges (Fig. 1c); tumor cells were observable at various mitotic stages (Fig. 1h, i, and j), cell-in-cell arrangement (Fig. 1k) and epithelial like cell distribution were occasionally observed (Fig. 1d). We also observed rimmed structural features in malignant serosal effusion (Fig. 1l), *i*.*e*., many tumor cells were distributed in ring-like structure, with giant tumor cells in the center. We also observed mulberry-like structures in which a tumor cell was surrounded by activated and aggregated lymphocytes (Fig. 1m), or tumor cell clusters were surrounded by a great number of lymphocytes (Fig. 1n).
2. Squamous cell carcinoma (Fig. 1o-q): scattered distribution; intensely stained cytoplasm; pink stained keratinized cytoplasm in some cells accompanied by shrunk nuclei (Fig. 1o); intensely stained, flocculated or granular chromatin, some multi-nucleated cells (Fig. 1p); small red-staining nucleoli in most cells located toward the edge of nucleus (Fig. 1p/q).
3. Small-cell lung cancer (Fig. 1r-s): mostly clustered; smaller than other types of tumor cells; greatly increased nucleo-cytoplasmic ratio, nuclei appearing as naked nuclei; single nucleus in most cells; intensely stained coarse granular chromatin; nucleoli not clearly visible.
4. Hepatocellular carcinoma (Fig. 1t-u): scattered distribution, regular shape; clearly defined boundary; intensely stained cytoplasm; intensely and uniformly stained chromatin; a single large, round, and bright red-staining nucleolus found in each of the center of nuclei.
5. Large-cell lung cancer (Fig. 1v-x): scattered distribution; boundary not clearly defined; giant cells (Fig. 1v); single nucleus in most cells; few multi-nuclei cells (Fig. 1w); pale stained cytoplasm with large nuclear-cytoplasimc ratio, nuclei appearing as naked nuclei; intensely stained coarse granular chromatin; obscure red-staining nucleoli in some cells with smaller size (Fig. 1v/x).
6. Malignant melanoma (Fig. 1y-z): scattered or clustered distribution; pseudopodia-like membrane protrusions at the cell periphery; intensely stained nuclei and cytoplasm; some cells multi- nucleolus (Fig. 1y); red-staining nucleolus with irregular size found in the nucleus; some cells multi-nucleated (Fig. 1y); presence of black granular substances in irregular size in the nucleus or cytoplasm (Fig. 1z).

### Results from the prospective, comparative study between the Thinprep and Cytospin-WG methods

#### Detection rate of cancer cells in CSF

In this comparative study, the initial positive rate of the Thinprep method was significantly higher than that the Cytospin-WG method [73.3% (33/45) vs. 57.8% (26/45); *P* = 0.016]. Further comparisons revealed the following outcomes: 1). Twenty six patients were positive for both the Thinprep and Cytospin-WG methods; 2) Twelve patients who were negative for the Thinprep method were also negative for the Cytospin-WG method, including 6 adenocarcinomas, 1 squamous cell carcinoma, and 5 small-cell lung carcinomas; and 3) seven patients (3 adenocarcinomas, 1 squamous cell carcinoma, and 3 small-cell lung carcinomas) having positive results from the Thinprep method were negative for the Cytospin-WG method. These seven patients shared a common feature for both methods in that only a small number of malignant cells were detected in their CSF samples. Among them, two (28.6%) showed scattered small clusters of malignant cells, and five (71.4%) showed scattered single malignant cells.

#### Morphology

**Fig 2:**
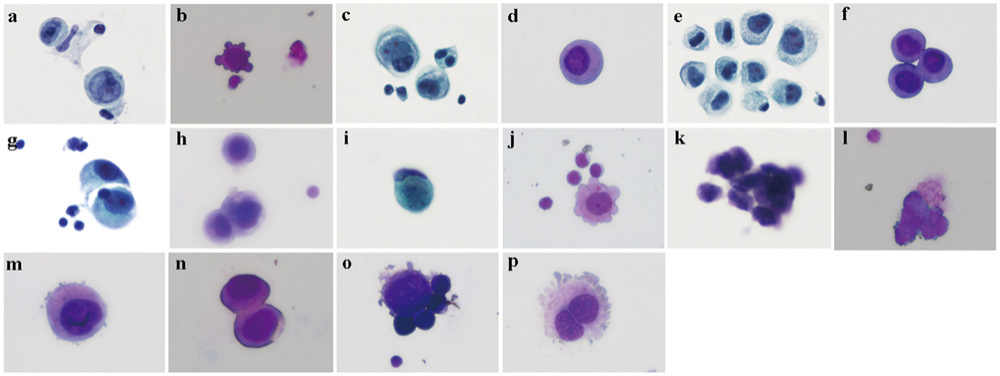
Side-by-side comparisons of CSF cytology results between the Thinprep plus Papanicolaou stain method (a, c, e, g, i and k) and the Cytospin-WG method (b, d, f, h, j and l) in patients with LM from various solid tumors. Representative slides from identical CSF samples are shown for the same samples using the two methods in paired photos: a and b, gastric adenocarcinoma origin; c-h, lung adenocarcinoma origin; i and j breast adenocarcinoma origin; k and l, small-cell lung cancer origin. m-p, the typical features of malignancy of tumor cells seen by the Cytospin- WG stain method.

In the comparative study, the typical features of malignancy such as large size, intense staining, and thickened nuclear membrane of tumor cells were visible in Cytospin-WG preparations (Fig. 2b, d, f, h, j, and l). Abnormal nuclear division (Fig. 2m), cytoplasmic bridges (Fig. 2n), and tumor cells encircled by lymphocytes were also occasionally observed (Fig. 2o). However, compared with the Thinprep results, most chromatin and nucleoli were not presented with clarity (Fig. 2a-l). With the WGC method, cells appeared shrunk and deformed (Fig. 2b/d/f); but in the same subject with the Thinprep method, cells had a full, regular cell morphology (Fig. 2a/c/e). Nipple-like protrusions were commonly observed at the cell periphery with the WGC method (Fig. 2b and j), but not seen in the same subject with the Thinprep method (Fig. 2a and i). Many cells on the glass slide appeared degenerated or ruptured with the WGC method (Fig. 2b and l), but not with the Thinprep method (Fig. 2a and k). This may have resulted from air exposure during dry-fixing and slide preparation, which may be one of the reasons for the low diagnostic rate of Cytospin-WG.

During Cytospin preparation, cellular components may adhere to the filter paper, resulting in cell loss. Since tumor cells were randomly distributed on glass slides as single cells or in clusters, it would be difficult to determine their numbers and density. However, in CSF samples of equal volume, the number of tumor cells was remarkably different between patients, indicating different tumor cell density between patients. In the comparative study, samples prepared by Cytospin method had a significantly fewer number of tumor cells than those prepared by the Thinprep method. For example, in a case of breast cancer, only two tumor cells were detected in one out of three slides prepared by Cytospin method, whilst over 10 tumor cells were detected in a single slide prepared by the Thinprep method (Fig. 2i-j). In samples that tested negative in Cytospin but positive in Thinprep, only a small number of tumor cells were detected in Thinprep, either as single cells or in small clusters. In these samples, either no abnormal cells were detected in slides prepared by Cytospin, or there were few abnormal cells, but they could not be definitively categorized as tumor cells for the lack of clear presentation of chromatin and nucleoli and other malignant characteristics.

The WG staining is more suitable for hematopoietic cell staining, and is one of the best staining methods for blood and bone marrow smears. Historically, LM diagnosis and treatment have mostly been focused on LM from hematologic malignancies, especially meningeal leukemia. Therefore, at present, the WG stain is routinely used in many institutions in China for CSF cytological examination. Results in this study showed that most solid tumor cells were stained pink or purple-red by the WG staining, and some nucleoli appeared gray in a purple-red background(Fig. 2p), or intensely stained in blue-purple with obscure presentation (Fig. 2d/j). The cellular structures were not presented with sufficient clarity. Only a few samples were stained with clarity (Fig. 2p). In summary, the Cytospin method was less sensitive than the Thinprep in cell collection, which may be one of the major factors causing false negative results, especially in patients who had a low tumor cell density in CSF.

The Pap staining has long been recognized as a suitable stain for malignant tumors of epithelial origin such as cervical cancer and urinary tract tumors [12]. Since solid tumor-caused LM often originates from primary tumors of epithelial origin, Pap staining may be a more appropriate staining method for CSF specimens from these patients. Results from this study showed that in most tumor cells stained by Pap stain, the cytoplasm was stained blue or blue-green; nuclear chromatin was stained blue-purple or dark blue; and nucleoli were stained red or purple. The structural features of chromatin and nucleoli were also presented with clarity. Cellular structures were presented with high clarity and high contrast. Various images of abnormal nuclear divisions and the process of cell division were observed with clarity.

## Discussion

Detection of tumor cells in CSF is regarded as the gold standard for diagnosing LM derived from solid tumors[1]. The detection of malignant cells is primarily based on the cell morphology (that significantly differs from normal cells), along with specific features only seen in malignant cells.

The Cytospin-WG method is commonly used in CSF cytological studies. However, this method is not ideal for detection of malignant cells in CSF from solid tumors because it does not provide clear presentation of morphological features and subcellular structures. The Thinprep method is a relatively new cytology method that has not been used as widely as the cytospin method in the clinical settings. More importantly, the morphological features of solid tumor-derived malignant cells in CSF specimens using the Thinprep method have not been reported. In this study, we performed cytological analysis of CSF specimens from patients with LM from solid tumors using the Thinprep method, and identified morphological features of malignant cells of specific pathological types. To the best of our knowledge, this is the first report on the CSF cytology describing morphological features of tumor cells of specific cancer types, using the Thinprep method.

We found that tumor cells processed with the Thinprep method had very clear presentation of 3D-like cellular structures. Our results indicated that each type of tumor cell in the CSF samples had specific identifiable morphological features. We then compared the diagnostic performance of the Thinprep method with the conventional Cytospin-WG method. Our results showed that the detection rate of LM by the former was significantly higher than the latter, which may be attributed to more efficient cell transfer, better preservation, and better presentation with the Thinprep method and Pap staining. Based on our results, we believe that cell damage and cell loss can be attributed to slide preparation and fixation, which often obscures the presentation of chromatin and nucleoli by the WG staining and may be the main reasons for the low detection rate of Cytospin-WG in detecting solid tumor cells in CFS specimens.

Although CSF cytology is often used for detection of malignancy, it is rarely employed in differential diagnosis of cancer types. [4] The primary tumor types are mainly determined by histologic examinations of primary cancers. However, for patients who are diagnosed with LM with unknown primary tumor at the time of LM diagnosis, examination of tumor cells by CSF cytology may provide clues for identifying the primary tumor. It may also help design appropriate chemotherapy for LM patients whose primary tumors are not identified at the time of treatment.

During the entire process of Thinprep method, cells were kept in their original liquid environment where they are likely to be protected from cataplasia caused by air exposure, which is likely to be the underlying reason for the well-preserved structural features of the cells seen in our studies. Microscopically, cells appeared to have rich and integrated 3D-like structures revealed by high-contrast staining. Fine structural details were visible in the chromatin and nucleoli. We found that morphological features of tumor cells are pathological type specific. Our results demonstrated that the initial diagnosis rate by Thinprep was 73.3% (33/45), which is much greater than a 40–50% initial diagnosis rate of solid tumor-caused LM by CSF cytology reported by some previous studies [13,14]. We believe the diagnosis rate may be improved by increasing the CSF sample volume [15].

## Conclusion

In the present study, we have demonstrated that Thinprep method provides, good preservation of cell morphology, and clearer presentation of subcellular structures. The Thinprep method has higher sensitivity compared to the Cytospin-WG method. In addition, the Thinprep may be used to differentiate tumor cell types in CSF specimens, which may improve the early differential diagnosis of LM in CSF and identification of cell type of the primary tumors, thus providing guidance for early therapy of patients with LM from various solid tumors.

## References

[1] Le Rhun E, Taillibert S, Chamberlain MC. Carcinomatous meningitis: Leptomeningeal metastases in solid tumors. Surg Neurol Int. 2013; 4: S265–288. 10.4103/2152-7806.111304 23717798 PMC3656567

[2] Straathof CS, de Bruin HG, Dippel DW, Vecht CJ. The diagnostic accuracy of magnetic resonance imaging and cerebrospinal fluid cytology in leptomeningeal metastasis. J Neurol. 1999; 246: 810–814. 10525979 10.1007/s004150050459

[3] Singh G, Mathur SR, Iyer VK, Jain D. Cytopathology of neoplastic meningitis: A series of 66 cases from a tertiary care center. Cytojournal. 2013; 10: 13 10.4103/1742-6413.114212 23858323 PMC3709425

[4] Prayson RA, Fischler DF. Cerebrospinal fluid cytology: an 11-year experience with 5951 specimens. Arch Pathol Lab Med. 1998; 122: 47–51. 9448016

[5] Argon A, Uyaroglu MA, Nart D, Veral A, Kitapcioglu G. The effectiveness of the liquid-based preparation method in cerebrospinal fluid cytology. Acta Cytol. 2013; 57: 266–270. 10.1159/000346716 23636078

[6] Weston CL, Glantz MJ, Connor JR. Detection of cancer cells in the cerebrospinal fluid: current methods and future directions. Fluids Barriers CNS. 2011; 8: 14 10.1186/2045-8118-8-14 21371327 PMC3059292

[7] Michael CW, McConnel J, Pecott J, Afify AM, Al-Khafaji B. Comparison of ThinPrep and TriPath PREP liquid-based preparations in nongynecologic specimens: a pilot study. Diagn Cytopathol. 2001; 25: 177–184. 11536442 10.1002/dc.2033

[8] Veneti S, Daskalopoulou D, Zervoudis S, Papasotiriou E, Ioannidou-Mouzaka L. Liquid-based cytology in breast fine needle aspiration. Comparison with the conventional smear. Acta Cytol. 2003; 47: 188–192. 12685187 10.1159/000326502

[9] Salhadar A, Massarini-Wafai R, Wojcik EM. Routine use of ThinPrep method in fine-needle aspiration material as an adjunct to standard smears. Diagn Cytopathol. 2001; 25: 101–103. 11477712 10.1002/dc.2012

[10] Ylagan LR, Zhai J. The value of ThinPrep and cytospin preparation in pleural effusion cytological diagnosis of mesothelioma and adenocarcinoma. Diagn Cytopathol. 2005; 32: 137–144. 15690333 10.1002/dc.20200

[11] Jan Drappatz M, Tracy T. Batchelor M, MPH. Leptomeningeal Metastasis ASCO education book. 2009: 100–105.

[12] Bristow RE, Montz FJ. Workup of the abnormal Pap test. Clin Cornerstone. 2000; 3: 12–24. 11061064 10.1016/s1098-3597(00)90018-8

[13] Chamberlain MC. Neoplastic meningitis. J Clin Oncol. 2005; 23: 3605–3613. 15908671 10.1200/JCO.2005.01.131

[14] Wasserstrom WR, Glass JP, Posner JB. Diagnosis and treatment of leptomeningeal metastases from solid tumors: experience with 90 patients. Cancer. 1982; 49: 759–772. 6895713 10.1002/1097-0142(19820215)49:4<759::aid-cncr2820490427>3.0.co;2-7

[15] Glantz MJ, Cole BF, Glantz LK, Cobb J, Mills P, Lekos A, et al Cerebrospinal fluid cytology in patients with cancer: minimizing false-negative results. Cancer. 1998; 82: 733–739. 9477107 10.1002/(sici)1097-0142(19980215)82:4<733::aid-cncr17>3.0.co;2-z

